# A Practical Guide to Using Time-and-Motion Methods to Monitor Compliance With Hand Hygiene Guidelines: Experience From Tanzanian Labor Wards

**DOI:** 10.9745/GHSP-D-20-00221

**Published:** 2020-12-23

**Authors:** Giorgia Gon, Said M. Ali, Robert Aunger, Oona M. Campbell, Mícheál de Barra, Marijn de Bruin, Mohammed Juma, Stephen Nash, Amour Tajo, Johanna Westbrook, Susannah Woodd, Wendy J. Graham

**Affiliations:** a London School of Hygiene and Tropical Medicine, London, United Kingdom.; b Public Health Laboratory-Ivo de Carneri, Pemba, Zanzibar, Tanzania.; c Brunel University London, Department of Life Sciences, Uxbridge, United Kingdom.; d Institute of Applied Health Sciences, University of Aberdeen, Aberdeen, United Kingdom.; e Department of IQ Healthcare, Radboud University Medical Center, Radboud Institute for Health Sciences, Nijmegen, The Netherlands.; f Macquarie University, Sydney, Australia.

## Abstract

Understanding hand hygiene behaviors is critical in hospitals. We developed the HANDS at birth tool—and provide information on its design and implementation–to capture the complex patterns of health care workers’ hand hygiene including hand rubbing/washing, glove use, and recontamination.

## Teaser

**Key Messages:**

- Time-and-motion methods are a good way of capturing hand hygiene compliance; for example, they can limit observer bias.
- We describe how we designed the HANDS at Birth tool, the tool format and its elements, its implementation components, the tool’s performance, and the implications for data analysis.
- The advantages of using this tool include simpler training, less observer judgment in assessing hand hygiene compliance, and improved ability to monitor multiple behaviors.

**Background:**

Good-quality evidence on hand hygiene compliance among birth attendants in low-resource labor wards is limited. The World Health Organization Hand Hygiene Observation Form is widely used for directly observing behaviors, but it does not support capturing complex patterns of behavior. We developed the HANDS at Birth tool for direct observational studies of complex patterns of hand rubbing/washing, glove use, recontamination, and their determinants among birth attendants. Understanding these behaviors is particularly critical in wards with variable patient volumes or unpredictable patient complications, such as emergency departments, operating wards, or triage and isolation wards during epidemics. Here we provide detailed information on the design and implementation of the HANDS at Birth tool, with a particular focus on low-resource settings. We developed the HANDS at Birth tool from available guidelines, unstructured observation, and iterative refinement based on consultation with collaborators and pilot results. We designed the tool with WOMBAT software, which supports collecting multidimensional time-and-motion data. Our analysis of the tool’s performance centered on interobserver agreement and convergent validity and the implications of the data structure for data analysis. The HANDS at Birth tool encompasses various hand actions and context-relevant information. Hand actions include procedures relevant during labor and delivery; hand hygiene or glove actions; and other types of touch. During field implementation, we used the tool for continuous observation of the birth attendant. Interobserver agreement was good (kappa range: 0.7–0.9), and the tool showed convergent validity. Using the HANDS at Birth tool is a feasible way to obtain useful information about compliance with hand hygiene procedures. The tool could be used after simple training and allows for collection of reliable information about the complex pattern of hygiene behaviors. Future studies should explore using this tool to observe behavior in labor wards in other settings and in other types of wards.

## BACKGROUND

Infection prevention is paramount to limiting the spread of epidemics, such as coronavirus disease 2019 (COVID-19), severe acute respiratory syndrome, and Ebola, and hand hygiene (HH) is at the forefront of prevention efforts among health care workers.^1^ In addition, health care workers’ HH is essential at the time of birth for preventing health care-associated infections that lead to an enormous burden of illness among mothers and newborns, even in nonepidemic situations.^2^ ^–^ ^4^ Accurately understanding the specific actions that contribute to the low compliance levels for HH that occurs in many countries, particularly in low-resource settings, is essential for effective behavior change; yet current tools fail to account for health care workers’ workflow and the issue of recontamination and its drivers.

Multiple methods exist to measure HH compliance in health care settings, but observation of behaviors is considered to be the gold standard.^5^ Observation can be done by an observer or by video recording. A recent validation study suggests that both approaches capture similar numbers of HH opportunities—moments when health care workers ought to practice hand rubbing/washing^6^; however, video recording poses substantial ethical issues, which often makes it difficult to use, particularly in a process such as childbirth when women are vulnerable and undressed. The World Health Organization (WHO) HH Observation Form is an excellent, widely used tool for direct observation.^7^ However, due to its aim and scope, it does not allow capturing more complex patterns of behavior. For example, it does not distinguish whether the failure to comply was because hand rubbing/washing was not attempted or because hands were recontaminated after initial washing.^8^ Avoiding hand/glove recontamination is implicit in the WHO tool’s HH definition because touching a surface carries the risk of germ transmission and creates a new HH opportunity. It also does not aim to capture the use or “misuse” of gloves.^9^ Finally, it requires the observers to judge when a new HH opportunity arises, thereby reducing the consistency of data collection by multiple observers.

Defining when a new HH opportunity arises is particularly difficult in labor and delivery, during which observers must deal with a transition from observing 1 patient (the mother) to 2 (mother and newborn). Furthermore, the amount, type, and location of body fluids can rapidly change during labor and delivery, and in the context of low-resource settings, a single health care worker may attend many mothers simultaneously. With an often unpredictable duration of the different stages of labor, the time between hand rubbing/washing and delivery of the newborn may be lengthy, during which time the observer needs to pay close attention to assess if any actions occur that lead to a new HH opportunity. Time-and-motion methods can overcome some of these challenges. These methods are now at the forefront of health care observation^10^ and are increasingly used, but seldom in low- and middle-income countries. These methods enable observers to record all health care workers’ actions without having to decide which ones represent a new HH opportunity. Instead, opportunities are defined during data analysis.

Time-and-motion methods can overcome some of the challenges with direct observation because they enable observers to record all health care workers’ actions without having to decide which ones represent a new hand hygiene opportunity.

The HANDS study (Hand-hygiene of Attendants for Newborn Deliveries and Survival) was a mixed-methods, cross-sectional research study conducted in the 10 highest volume maternity wards in Zanzibar between November 2015 and April 2017.^8^ The aim of the study was to explore compliance with HH guidelines and identify factors that explain compliance. HH during labor and delivery is a key opportunity to prevent transmitting infections to mothers and newborns^3^ ^,^ ^11^; however, good-quality evidence on HH compliance from low-resource labor wards is limited.^12^ ^–^ ^16^ Therefore, we developed the HANDS at Birth tool, based on a time-and-motion design, to observe the complex patterns of birth attendants’ HH and glove use at 3 levels: the opportunity, the individual, and the facility. We designed the tool within WOMBAT software, which is intended to support direct observational studies of health care work. The WOMBAT software package^17^ ^,^ ^18^ allows collecting multidimensional work tasks, including compliance with specific tasks, and automatically time-stamps data entry. The current investigation was one of the few time-and-motion studies of health care workers conducted with software that automatically records time and carried out in a low-resource setting.^19^ ^,^ ^20^

Our aim was to provide very practical details regarding the design and implementation of the direct observational tool to measure HH compliance to inform researchers and practitioners seeking to thoroughly measure the compliance with HH guidelines during labor and delivery, particularly in low-resource settings. In this article, we outline: (1) how we designed the data collection tool, (2) the tool format and its elements, (3) its implementation components, (4) the tool’s performance, and (5) the implications for data analysis.

## METHODS FOR TOOL DEVELOPMENT

We developed the HANDS at Birth data collection tool between March and October 2016 using an existing systematic process for tool development.^13^ This process included use of available guidelines, unstructured observation, and iterative refinement based on consultation with collaborators and pilot results.

### Guidelines’ Review and Semistructured Observation

We consulted WHO publications, including *Hand Hygiene Technical Reference Manual*,^7^ *Hand Hygiene in Outpatient and Home-Based Care and Long-term Care Facilities*,^21^ and *Pregnancy, Childbirth, Post-partum and Newborn Care: A Guide for Essential Practice*.^22^ We also conducted 11 semi-structured observation sessions in 4 labor wards in Zanzibar during which either a delivery or a vaginal examination occurred. All birth attendants’ actions were recorded, together with the time when they happened and their location. Using this information, we created a list of procedures (what we also call “key attendant-patient interactions”) relevant to labor and delivery that also included other hand actions that can occur before and after each of these procedures.

### Iterative Collaborator Consultation

The project was a partnership of the London School of Hygiene and Tropical Medicine, the University of Aberdeen, and the Public Health Laboratory of Pemba; we sought feedback on the tool from all project members. Additionally, a 3-hour in-depth consultation was conducted with 2 clinically trained members of the team (1 general practitioner and 1 midwife) who provided additional feedback.

### Pilot Activities and Training

We conducted 3 pilot activities in a labor ward on Pemba Island, Zanzibar, Tanzania. Two data collectors conducted the first pilot in June 2016 using an early version of the HANDS at Birth tool. One data collector conducted the second pilot in August 2016 using the tool incorporated into WOMBAT v2 software on a tablet. Finally, 1 data collector conducted the third pilot in September 2016 using the tool with WOMBAT. Feedback was collected and incorporated to improve the tool at each stage.

Observers were trained to use the tool over 3 days using role-plays and presentations. Each observer also practiced using the tool in the labor ward for 3 hours under trainer supervision (GG). The trainer also conducted 2 hours of observation with each observer and provided them with relevant feedback. During training, minor refinements were made to the tool.

We used the STROBE checklist for cross-sectional studies to design and describe this tool here and in other relevant manuscripts including the study results.^23^

The project was approved by the Zanzibar Medical Research and Ethics Committee, the London School of Hygiene and Tropical Medicine Research Ethics Committee, and the Research Ethics Committee at the University of Aberdeen. Details of procedures to consent are described below.

## TOOL FORMAT AND ELEMENTS

Following Lopetegui et al.’s classification,^10^ our time-and-motion study used continuous observation, in which an external observer focuses on 1 subject, in our case, the birth attendant. When a birth attendant performed an action, the observer recorded the action. We chose to use continuous observation because the timing of procedures, particularly delivery itself, was typically unpredictable, and using alternative methods, such as short observation sessions at fixed or random intervals, could have missed many HH opportunities. Hence, observers were asked to remain in the labor room for the entirety of their allocated shift (about 7 hours for morning/afternoon shifts and 10 hours for night shifts) and to start recording observations whenever a patient-attendant interaction began.

Our time-and-motion study used continuous observation, in which an external observer focuses on one subject, such as a birth attendant.

The tool, available in Supplement 1, includes a list of hand actions and context-relevant information (Figure). The hand actions listed were exhaustive (meaning that the list did not leave any possible actions out) and mutually exclusive (meaning that no 2 actions could occur simultaneously). We did not design a tool that aimed to capture multitasking or interruptions because we did not want to add to the burden on the observers.

**FIGURE:**
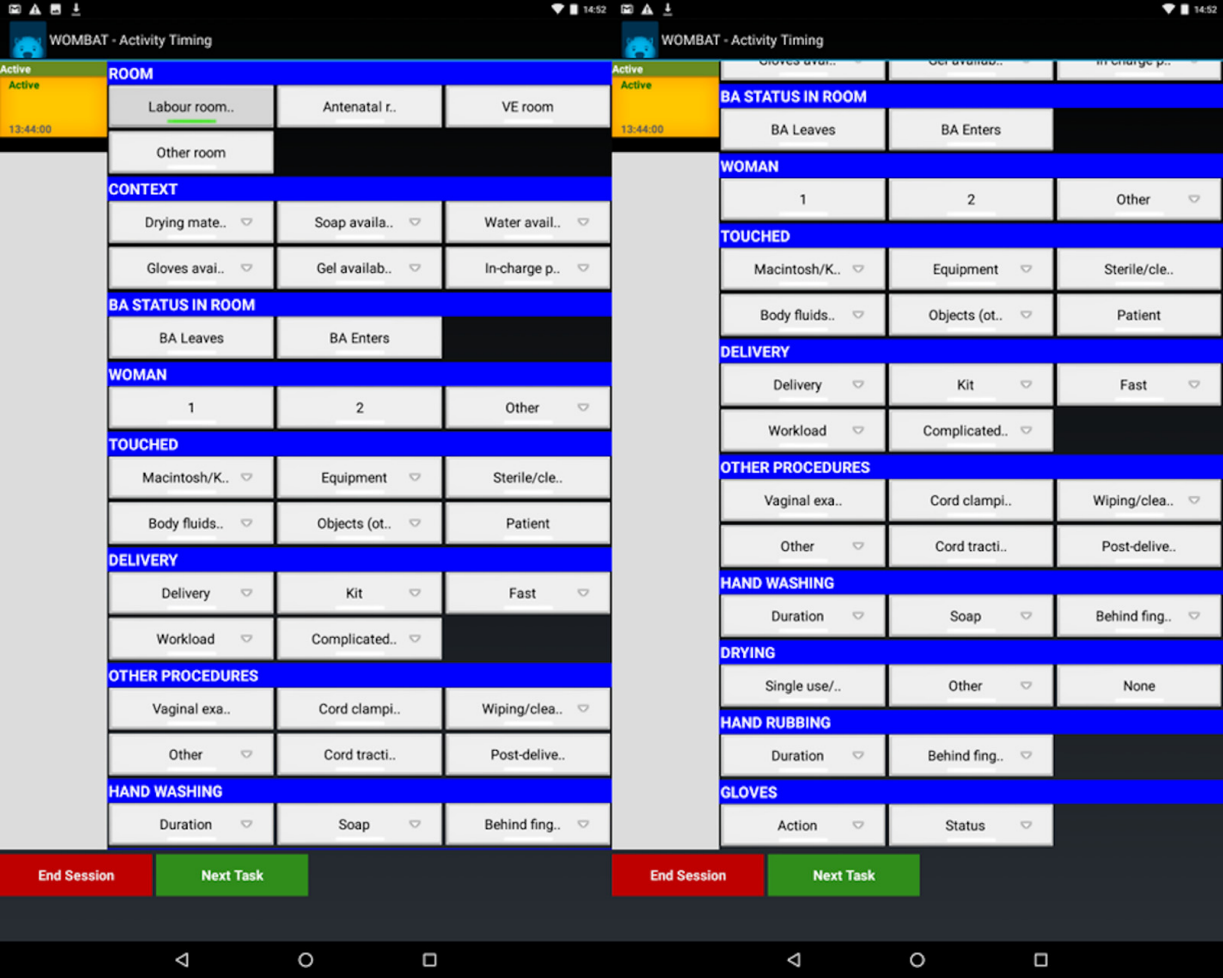
Screen Showing HANDS at Birth Tool to Collect Multidimensional Time-and-Motion Data on Hand Actions and Context-Relevant Information on Hand Hygiene. (Left) Screen That Appears When User Logs In to Tool. (Right) Screen That Appears When User Scrolls Down. Abbreviations: BA, birth attendant; VE, vaginal examination.

Hand actions were either procedures relevant during labor and delivery (e.g., vaginal examination) (Table 1), HH or glove actions, or other types of touches (e.g., touching a pen or equipment). Observers recorded when an attendant left the room where observation was occurring (when observation was suspended) and when the attendant re-entered.

**TABLE 1.** Relevant Hand Actions During Labor and Delivery Included in the HANDS at Birth Tool for Observation of Birth Attendants

Measuring vital signs
Wiping the vagina
Vaginal examination
Artificial rupture of membranes
Episiotomy
Catching the baby (delivery)
Cord cutting and clamping
Cord traction
Postdelivery examination of the vagina
Wiping the baby clean after birth
Supporting breastfeeding
Manual removal of placenta
Suturing
Suctioning baby’s nose/mouth
Using bag and mask on the baby
Catheter insertion or removal
Insertion or removal of IV lines
Adjusting IV fluids or changing IV bag

Abbreviation: IV, intravenous.

The tool also captured information on the context, such as availability of key infrastructure/staffing (e.g., water or the presence of the nurse in-charge) and which woman was being attended (first, second, third, etc. since the beginning of the observation session). This process allowed us to assess whether birth attendants performed HH between patients. Observers entered this context-related information at the beginning of the observation session and updated it only if the situation changed.

Many of the recorded actions required further details to be entered. For example, when a delivery was observed, the observer also recorded whether the delivery occurred rapidly (within 5 minutes of the woman walking into the labor room), whether there were complications, whether the observer birth attendant had an assistant, and whether a premade delivery kit was used. The observers collected contextual information and details of certain actions because we intended to use these data as potential determinants of HH in the analysis. The determinants collected and associated with HH are described in detail in Gon et al.^24^

## TOOL IMPLEMENTATION

This section characterizes how we used the tool to collect data and provides considerations for using it in future studies. We used the guidance provided by Zheng et al.^25^ for reporting time-and-motion studies, and include their full STAMP checklist information in Supplement 2.

### Sample Size Calculations

The data collection timeframe was based on the expected number of deliveries in the targeted facilities. We estimated the latter, using the formula for estimating a proportion from a cross-sectional survey with α=0.05 and 80% power. We used a design effect of 2 based on a survey by Rowe et al.^26^ To estimate a hand rubbing/washing compliance of 10% with an absolute precision of ±3%, we needed 768 HH opportunities. We estimated the length of observation needed to collect this number, and in practice these data were collected during 336 observation sessions ranging from 13 minutes to 6 hours 45 minutes, with a median time of 1 hour and 41 minutes.^8^ As described in Gon et al,^8^ we collected information on 781 HH opportunities before aseptic procedures (*before aseptic procedures* is 1 of the 5 types of HH opportunity prescribed by WHO^7^).

### Planning and Logistics of Data Collection

To obtain representative data on deliveries across all shifts (morning, afternoon, and night); 3 observers, 1 per shift, conducted observations that covered 24 hour a day. They observed for a total of 130 hours in the morning, 153 hours in the afternoon, and 205 hours in the night. Each observer had their own tablet for data collection. Each facility was visited for a mode of 6 consecutive days (range: 5–14 days) between September 17 and December 31, 2016. The order in which we visited the facilities was based on logistics. We arranged for additional days of observation in 1 facility with a high volume of staff to allow all staff to be observed and in 3 facilities with low volume of deliveries to capture a sufficient number of procedures.

We consulted the ward rosters to allocate individual attendants to the observers. Each attendant had a unique identifier that the observer had to record in WOMBAT when observing them. Observers were allocated to shifts based on the following principles: (1) the same observer should observe the same attendant so the attendant becomes accustomed to the same person being on the ward; (2) the initial attendant-observer pairs at each facility were assigned at random (unless specific concerns were raised; e.g., some flexibility on choice of types of shifts was allowed to accommodate observers’ needs); and (3) observation days should ideally be planned during changes in shift pattern to allow observation of the same attendants working on different types of shifts. The need to observe the same attendant across different types of shifts using the same observer increased the fieldwork duration and therefore had to be counterbalanced by the need to remain within our budget.

### The Observers

Observers were all trained nurse-midwives working in managerial roles. Two of them worked in the study facilities but not in the labor wards. The third observer worked in district-level management. Their previous knowledge and understanding of the labor process were vital to ensuring quality during data collection and ultimately the project’s success.

Observers were all trained nurse-midwives, whose previous knowledge and understanding of the labor process were vital to the success of the project.

### Study Participants

All birth attendants present during the observation period who were involved in the childbirth procedures outlined in Table 1 were eligible for observation. We observed a total of 104 birth attendants across the 10 facilities and between 4 and 15 birth attendants in each facility. Each attendant was observed for 1–9 observation sessions.^8^ In each observation session, only 1 attendant was observed, but that attendant could be caring for multiple women and carrying out many procedures. Attendants in our study were all women, 90% were professionally trained, and 10% were health orderlies/nonprofessionals.

The attendants’ responsibilities were usually allocated during the shift itself. We encouraged observers to listen at staff meetings to learn which attendant was most likely to perform the childbirth procedures outlined in Table 1 to decide whom to observe. Observers were instructed to observe each allocated birth attendant roughly equally in each facility.

### How to Observe

We trained the observers to enter only 1 action at a time to facilitate the data input process. We were specifically interested in the attendants’ actions, the sequence of these actions, and the length of time between them, rather than the duration of each action per se. An action was selected and entered immediately. We do not have details on when the action ended, but since the actions were mutually exclusive, it was clear when one action replaced another.

We were specifically interested in the attendants’ actions, the sequence of these actions, and the length of time between them.

### When to Observe

As described above, a relevant patient-attendant interaction (Table 1) triggered the start of data entry; observers were expected to be continuously present in the ward due to the unpredictable nature of birth. Observers were encouraged to take breaks when no women were in labor or when women were in very early stages of labor and to remain where they could see if an emergency admission occurred to avoid missing delivery events. We also encouraged breaks if the observer’s concentration level was low.

We instructed observers to end a session when a major procedure ended and no further patient activities were in sight, when the observer wanted to take a break, when there was the opportunity to start observing another birth attendant, or when the birth attendants left the room to perform duties elsewhere.

### Where to Observe

Observers would usually sit in the labor room. If no deliveries were happening, we asked observers to observe vaginal examinations in other rooms, such as the antenatal ward or examination room.

### Consent and Study Aim Concealment

Written consent was gathered from women in the antenatal ward before observation; alternatively, women were asked for verbal consent once in the labor ward, and follow-up for written consent occurred in the postnatal ward before discharge or before delivery in the antenatal ward.^8^ Women were told that no demographic information was collected on them and recorded observations were exclusively regarding birth attendants’ behavior. Permission to observe the attendants was obtained by the Ministry of Health and verbal consent was obtained by the observers when they first visited the facility.^8^

Attendants were told the observation was about the quality of care at birth, not on HH specifically, to conceal the study’s focus and reduce the Hawthorne effect. In all but the facility in Zanzibar where piloting took place, the focus of the study (HH practices) was likely to have been well concealed from the birth attendants being observed. The pilot facility in Zanzibar had the highest compliance with hand rubbing/washing before aseptic procedures. Compliance was 10% higher than the second-best facility and 7 times higher than the worst one. For ethical reasons, observers were trained to notify health workers and the field manager if they observed a potentially harmful condition or practice.

### Quality of Data Collection

To ensure quality of data collection, we held regular meetings with collectors by telephone and onsite, communicated via a WhatsApp group, held Skype calls at the end of observations in each facility, and monitored the data uploaded monthly. These communication channels enabled rapid feedback, answers to questions, and maintenance of morale during long periods of observation. Drivers ensured observers arrived at sites on time. Finally, we are confident that the data were unlikely to have been manufactured because manufacturing time-stamped data would require as much time as conducting and recording actual observations.

### Software and Information Technology Costs

The cost of the software and hardware also needs to be considered especially for deployment in low- and middle-income countries. WOMBAT 3.0 is available from the Apple Store (https://apps.apple.com/us/app/wombat-3-0/id1445107457). Data hosting is available at a cost of US$2,500 for a 2-year period, which allows the use of the software for multiple projects and data collectors. Free packages such as Open Data Kit could be used, but Open Data Kit is less user friendly for time-and-motion studies. In addition, we bought 3 tablets for approximately US$500.

## TOOL PERFORMANCE

### Interobserver (Interrater) Agreement

To report on interobserver agreement procedures and findings, we followed the recommendations by Lopetegui et al.^27^ for time-and-motion studies and consulted the WOMBAT guidelines.^18^ ^,^ ^28^ While piloting the tool, the trainer conducted 2 hours of simultaneous observation between the trainer (GG) and each of the observers. We then verified the extent of agreement between GG and each of the 3 observers on the basis of 28, 29, and 36 opportunities for hand washing/rubbing, glove wearing, and touch events, respectively. The observations were based on a total of 11 vaginal examinations and 5 deliveries. The exercise was also used to provide feedback to the observers.

During the first month of data collection, we also assessed interobserver agreement, whereby a pair of observers was allocated to 2 of the same shifts in the busiest facility and asked to observe the same attendants. Observers were asked to perform this independently, avoiding communication or looking at each other’s tablet, but we could not ensure they were blinded, which meant that they probably knew we were going to check the data and hence some form of communication might still have occurred. Two pairs carried out this exercise for 1 morning and 1 afternoon shift each, the other pair for 2 night shifts. Two pairs observed 3 birth attendants, and the third pair observed 4.

We calculated kappa statistics based on either 49 or 50 hand rubbing/washing, hand recontamination, or glove behaviors per pair of observers. Observations were based on a total of 9 vaginal examinations and 11 deliveries. Through visual inspection of the data, we ensured that the behaviors compared were the same between observers by checking the reported time and sequence of actions. The kappa statistic calculated for pairs of observers was good for 2 of the 3 pairs at 0.93 and 0.90, but it was below the optimal level of 0.85 for 1 of the pairs at 0.73.^18^ In addition, we are also confident that discrepancies between observers was minimal because our results showed that hand rubbing/washing compliance before aseptic procedures did not vary substantially by observer, as described in Gon et al.^8^

### Convergent Validity

We assessed the degree to which 2 measures of constructs that theoretically should be related were in fact related (convergent validity) by showing whether hand rubbing/washing before aseptic procedures compliance varied in the expected direction by contextual characteristics. Using the methods described in Gon et al,^24^ we descriptively showed that higher compliance was present when the necessary equipment (water and soap or gel) was available, when fewer women were attended in the same observation session (i.e., a lower workload was expected to be associated with better HH), and when attendants had received HH refresher training in the previous year (Table 2).

**TABLE 2.** Hand Rubbing/Washing Compliance Before Aseptic Procedures Among Birth Attendants in Health Facilities in Zanzibar, Tanzania

	Observed Opportunities/Indications for Hand Hygiene, per Guidelines^a^ n (%), N=779	Hand Hygiene Compliance(Hands Rubbed/Washed) When Indicated n (%), N=190
**Necessary hand hygiene equipment (water and soap or gel)**		
No	48 (6.2)	5 (10.4)
Yes	704 (90.4)	177 (25.1)
Missing	13 (1.7)	3 (23.1)
Inconsistent information	14 (1.8)	5 (35.7)
**Maximum number of women attended in an observation session**		
1	541 (69.5)	146 (27.0)
2	196 (25.2)	39 (19.9)
3	36 (4.6)	4 (11.1)
Missing	6 (0.8)	1 (16.7)
**Hand hygiene refresher training in the past 12 months**		
No	347 (44.5)	74 (21.3)
Yes	432 (55.5)	116 (26.9)

a Number of times when hand hygiene was meant to be performed per guidelines.

## IMPLICATIONS FOR DATA ANALYSIS AND INTERPRETATION

In Supplement 3, we describe data cleaning, analysis, and interpretation issues that needed to be considered, noting in particular, that some data items relied on observer subjectivity (e.g., duration of hand washing) and some variables (e.g., variables describing the context) required more stringent training than others.

### Data Structure

A strength of WOMBAT is that when each action is recorded, the time of that action is automatically logged. Our final dataset was a list of 7,893 time-ordered entries. These data were coded to derive HH opportunities and to calculate compliance. First, each HH opportunity needed to be identified within each observation session, which is further explained below. Second, for HH opportunities **before** aseptic procedures or touching the patient, the sequence of actions preceding the opportunity needed to be examined for hand rubbing/washing actions, glove use and actions that may lead to a new HH opportunity. Whereas, for HH opportunities **after** exposure to body fluids or touching the patient or the patient’s surrounding, the actions following the opportunity needed to be examined. We used STATA to analyze these data.

A strength of WOMBAT is that when each action is recorded, the time of that action is automatically logged.

### Time Stamps

We used WOMBAT’s time stamp information in 2 ways. First, we checked the plausibility of certain actions being linked; for example, a hand rubbing/washing action could not be linked to a procedure conducted 10 hours before or after it. Second, we calculated the length of time between hand rubbing/washing and the HH opportunity to determine whether time would predict the likelihood of hand recontamination occurring.

### A Priori Definitions Required

To estimate HH compliance, we operationalized definitions for the **systematic flow** of patient contacts allowed within a given HH opportunity and the **patient zone**. By a systematic flow, which we called a “delivery flow,”^8^ we referred to the procedures or actions of interest that defined the start of a new HH opportunity, as well as the sequence of these procedures, which occurred without a break and were considered as 1 opportunity for HH.^21^ For example, in a given delivery flow, a vaginal examination could be followed by the delivery of the baby, but not by touching a patient’s shoulder. During a delivery flow, a birth attendant could undertake hand actions within the patient zone without the need for a new HH opportunity to arise.

In this study, we defined patient zone as encompassing a woman’s perineal area and thighs, any clean or sterile equipment being used, and the newborn as it was caught and wiped. A break in the delivery flow, indicating a new HH opportunity, arose if an activity occurred that was outside the patient zone, such as inserting an intravenous line, touching the patient beyond the zone, or leaving the room.^8^

Details on the definitions used in our study are reported in Gon et al.^8^ Potentially, a separate software could be programmed to automatically analyze this type of data in the future, allowing for definitions to be applied from the outset.

### Context-Specific Adaptations

To classify which surfaces we should include in the patient zone, we used previous formative research^29^ on the microbiological load of the labor surfaces in Zanzibar, as well as unstructured observation of labor wards conducted within the HANDS project. For example, we excluded the delivery bed and trolley from the patient zone because previous work found that these surfaces were often contaminated with potential pathogens.^8^ Other important information to consider include the details of the cloth or plastic sheet used under the woman’s body during birth, the cleaning routines of the wards, the type of water available, the delivery equipment preparation, and the local HH guidelines against which to measure hand washing/rubbing duration and technique. It is not clear that all projects will have the capacity to gather this level of contextual information; however, capturing the real workflows in this context was our aim.

Ideally, all definitions should be clear at the start of a project, but during data collection, the project may accrue context-specific information on the surfaces or the attendants’ workflows, which should be used to update the definitions. To illustrate this, we present the number of HH opportunities and hand rubbing/washing compliance results for 4 different patient zone definitions (Supplement 4).

## DISCUSSION

We developed the HANDS at Birth tool to capture the complex HH and glove behaviors of birth attendants, based on state-of-the-art methods: a time-and-motion study using a computerized system (WOMBAT). This approach has been rarely used to measure HH or to conduct research in low-resource settings.^10^ ^,^ ^19^ ^,^ ^20^ ^,^ ^30^ ^,^ ^31^ Our time-and-motion study enabled us to accomplish the following, which would have not been possible with the WHO HH Observation Form: (1) to look at whether birth attendants comply with the complete sequence of behaviors prescribed by the WHO guidelines,^32^ (2) to look at each behavior individually, and (3) to look at different behavior sequences.^8^ Additionally, our method reduced the risk of observer bias because data collection was coded as a series of individual actions rather than relying on observer judgment that a new HH opportunity had occurred; hence, opportunities were identified retrospectively in a standardized way.^33^ Indeed, hand rubbing/washing compliance was similar between observers in our study, as reported in Gon et al.^8^ Beyond HH, the HANDS at Birth tool allowed investigation of other behavior sequences and workflows.

Our method reduced the risk of observer bias because data collection was coded as individual actions rather than relying on observer judgment that a new HH opportunity had occurred.

We are aware of 1 other study that used time-and-motion methods to report HH of health care workers in the context of an intensive care unit in the United States.^31^ That study’s aims differed from ours including determining the number of contacts between patients and health care workers, as well as how long they take, and estimating HH compliance specifically before entering a room and after exiting a room. That study did not detail information on the tool format or content. In comparison, the HANDS at Birth tool allows for a more exhaustive list of actions to be recorded, including those beyond patient-attendant interactions; it also allows looking at all HH opportunities, not just those related to exiting or entering the room.

This tool has the potential to be adapted to examine HH in other types of wards. We think this detailed examination of HH, including recontamination, is particularly important in wards facing unpredictable volumes of patients or unpredictable patient complications. Examples include emergency departments, operating wards, or isolation wards during epidemics, such as the current isolation wards for COVID-19 patients. In particular during the COVID-19 pandemic, this tool could lend itself to examining the key relationship between hands and surfaces and the fundamental issue of pathogen cross-contamination between them.^34^ ^,^ ^35^

### Limitations

Because we were interested in individual determinants of HH behavior, we observed only 1 birth attendant at any 1 time; whereas, the WHO HH Observation Form audit tool is designed to observe multiple health care workers simultaneously, which allows collection of more HH opportunities in the same observation session. Importantly, the HANDS at Birth tool is not intended to substitute for the WHO HH Observation Form; the 2 tools serve very different purposes, with the former being aimed at research and the latter at infection prevention practitioners. Another limitation of our tool, and how we used it, is that it requires data cleaning and data management. For example, even though misclassification was minimal, some actions were recorded by mistake at the same time. In addition, a couple of variables relied on observer subjectivity—for example whether a delivery happened very fast after the woman’s admission in the labor room. The structure of the data implies that data management is needed to create HH opportunities and HH compliance results.

## CONCLUSION

In conclusion, we report the process of developing a research tool to capture the complexity of HH and glove behavior during labor and delivery, including the tool elements, field implementation, tool performance, and implications for analysis. We used a computerized system that was feasible to use in low-resource facilities. Advantages of this tool include simpler training, less observer bias in assessing HH compliance (compared with the WHO HH Observation Form), and the ability to monitor multiple behaviors. The data it produced also showed good reliability and convergent validity. Future studies should explore the use of this research tool in labor wards in other contexts, as well as in other types of wards.

## Supplementary Material

20-00221-Gon-Supplement3.pdf

20-00221-Gon-Supplement4.pdf

20-00221-Gon-Supplement2.pdf

20-00221-Gon-Supplement1.pdf

## Notes

**Peer Reviewed**

**First published online:** October 15, 2020

**Funding:** The project was funded by the United Kingdom Research & Innovation Medical Research Council (MRC) Public Health Intervention Development scheme (award number MR/N015975/1). This award is jointly funded by the MRC and the United Kingdom Department for International Development (DFID) under the MRC/DFID Concordat agreement and is also part of the European & Developing Countries Clinical Trials Partnership program supported by the European Union. The Soapbox Collaborative also contributed by funding staff involved in this project.

**Data Availability:** The dataset generated during the current study is available at: https://doi.org/10.17037/DATA.00000778.
