# Supplementary material for: A Practical Guide to Using Time-and-Motion Methods to Monitor Compliance With Hand Hygiene Guidelines: Experience From Tanzanian Labor Wards
Source: Glob Health Sci Pract. 2020 Dec 23;8(4):827–37. doi: 10.9745/GHSP-D-20-00221 (PMC7784080; doi:10.9745/GHSP-D-20-00221)
Supplement: 20-00221-Gon-Supplement3.pdf [file 20-00221-Gon-Supplement3.pdf]

Supplement to: Gon G, Ali SM, Aunger R, et al. A practical guide to using time-and-motion methods to monitor compliance with hand hygiene guidelines: experience from Tanzanian labor wards. *Glob Health Sci Pract*. 2020;8(4). https://doi.org/10.9745/GHSP-D-20-00221

Supplement 3. Tool Items With Specific Implications for Data Cleaning, Analysis, and Interpretation

Items That Required Data Cleaning and Consistency Checks

The following is a list of mistakes in data entry that we found in our dataset and required data cleaning:

1. We performed consistency checks on action subcategories that were mutually exclusive. For example, if it was coded on the same entry that drying hands was carried out with single-use material and that no drying was performed, we could not tell the correct response, and the entry was recoded as "inconsistent information." "Inconsistent information" was not used in the analysis, and it was not very a frequent occurrence (<2% of data).
2. In a few cases, data collectors reported an action and its subcategories in successive separate entries, against the training instructions. For example, they would report a delivery, press enter, and then provide the detail of the delivery (e.g., type of delivery kits used or availability of an assistant) in one or multiple successive entries. Hence, we cleaned this data by allowing for the entry on the subcategories of an action to appear on the same line of entry as the action of interest.
3. In a few cases, multiple actions of different types were found on the same line of data entry; for example, a birth attendant touched a surface and at the same time that she assisted a delivery. Since we cannot make an assumption about which of the 2 actions happened first, we prioritized hand actions and procedures, which meant that some information was lost. This loss was minimal because the number of multiple actions entered simultaneously was small (37/7,893). Given the way we used the WOMBAT software, unless the data collector pressed enter after each action, it was possible to record 2 action simultaneously. Visual inspection of the data and conversation with data collectors suggests that data collectors, if they made a mistake, would sometimes repeat the recording of a certain action with the corrected information. However, we do not know how often this was done because we do not have video recording of the true situation to verify the counterfactuals. During the training, we could have had more practical exercises on how to revise the last action recorded to minimize this issue.
4. During cleaning, we also checked candidate data errors against field notes where possible. For example, one woman appeared to have triplets from the data. Since triplets are rare, we checked this information against the field notes and a triplet was indeed reported on the same day of the observation via the WhatsApp group.
5. In a few instances, the reported unavailability of a context-related item was contradicted by the performance of an action requiring that item. There were 14 instances when the data collector recorded unavailability of water but hand washing was performed. There were 10 occasions each when soap or alcohol hand gel were reported as not available but were also observed to be used. In these cases, we kept the observed behavior and changed the availability of the item to

"available" because behavior was monitored more constantly than the item availability. This questions further the reliability of the context-related data. It is important to note that we could check if the use of an item corresponded to its availability, but we could not check the opposite—if an item was not used. For example, if hand washing without soap was recorded, does it mean that soap was really there?

Items That Are More Affected by Observer Subjectivity

Two types of information relied on observer subjectivity: whether a delivery happened very fast after the woman entered the labor room and the duration of hand washing or rubbing. Neither item was timed—they were based on the data collectors' judgment. As per the training, a delivery was considered to be fast if the delivery of the baby occurred within 5 minutes of the woman entering the labor ward. Two of the 3 data collectors reported 70% of the deliveries they observed as "fast." One data collector (the one showing the highest qualitative agreement with the trainer) only recorded 20% of the deliveries observed as fast. This suggests that this indicator might not have been interpreted the same across all data collectors; we might have overestimated the frequency of fast deliveries that occurred in our sample. Among the times when hand washing or rubbing occurred, the 3 observers reported (Gon et al 2018) that the duration of hand washing or rubbing was more than 10 seconds in 8% (observer 1), 13% (observer 2), and 28% (observer 3) of these instances.

Because of the way we trained data collectors to use WOMBAT (i.e., focusing on tasks rather their duration) and because hand hygiene behaviors occur fast, we could not establish a simple way of timing the duration of hand washing or the delivery. Using a stopwatch or changing the way we used WOMBAT appeared too cumbersome for this group of data collectors and would have exacerbated observer biases. Some of the features of WOMBAT, such as its ability to capture task duration, could be exploited in future uses of this tool.

Items That Require Stringent Training

The variables describing the context (e.g., the availability of water) need updating when the environment changes compared with the status quo recorded at the beginning of the sessions. We have reason to think that data collectors sometimes forgot to update at least some of this context. For example, from data inspection and conversations with data collectors, it became evident halfway through data collection that 2 of the 3 data collectors did not always remember to update the information on whether the in-charge was present. In addition, information on the index number of the woman being attended could be improved: in 5 observations sessions, we did not have any index number recorded at all, indicating that this information was perhaps poorly recorded across the study. If there was no index number recorded or the number did not change during a session, we assumed during data analysis that the same woman was being assisted across a session. More tailored training could improve the data collection on these variables, or perhaps the use of a different software that reminds observers at regular intervals to update these variables.
