# Supplementary material for: A Practical Guide to Using Time-and-Motion Methods to Monitor Compliance With Hand Hygiene Guidelines: Experience From Tanzanian Labor Wards
Source: Glob Health Sci Pract. 2020 Dec 23;8(4):827–37. doi: 10.9745/GHSP-D-20-00221 (PMC7784080; doi:10.9745/GHSP-D-20-00221)
Supplement: 20-00221-Gon-Supplement4.pdf [file 20-00221-Gon-Supplement4.pdf]

Supplement to: Gon G, Ali SM, Aunger R, et al. A practical guide to using time-and-motion methods to monitor compliance with hand hygiene guidelines: experience from Tanzanian labor wards. *Glob Health Sci Pract*. 2020;8(4). https://doi.org/10.9745/GHSP-D-20-00221

Supplement 4. Changing the Definition of Patient Zone

Using the methods described in Gon et al. 2018, we calculated the compliance with hand washing/rubbing based on the following patient zone definition: the mother's perineum and thighs, newborn, and any other clean or neutral hand actions. To show how our tool performs with a different definition of the patient zone, we calculated the number of HH opportunities and the hand rubbing/washing compliance for an additional 3 patient zone scenarios described in the Figure below. Based on these results, the hand rubbing/washing compliance did not substantially change as the patient zone widened, although it increased slightly; whereas, the number of HH opportunities decreased as hypothesized.

Figure. Patient Zone Scenarios, Number of Opportunities, and Compliance (Green Highlights the Zone)

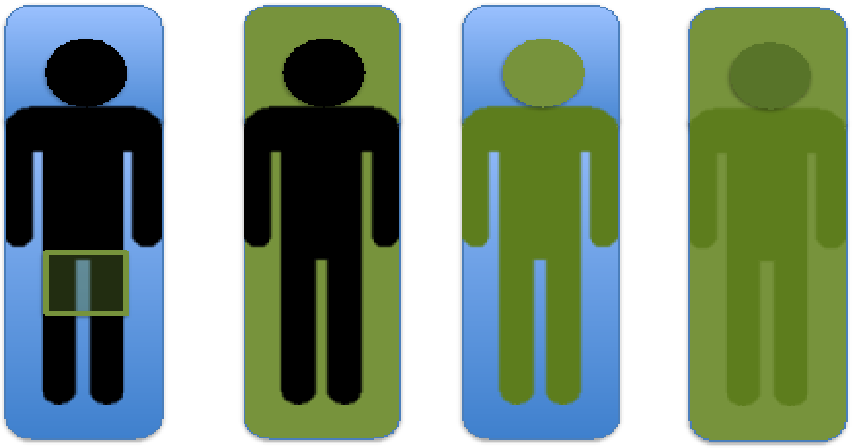

Patient zone 1	Patient zone 2	Patient zone 3	Patient zone 4	
Mother's perineum and thighs, newborn + clean or neutral hand actions	Zone 1 + unclean Macintosh + patient bed	Zone 1 + patient	Zone 1 + Zone 2 + Zone 3	**Definition of patient zone**
781	729	717	640	**Number of opportunities**
24.6 (21.6–27.8)	26.2 (23.0–29.6)	27.8 (23.7–30.2)	29.8 (26.3–33.6)	**Compliance to washing/rubbing, % (confidence interval)**
