# Supplementary material for: A Practical Guide to Using Time-and-Motion Methods to Monitor Compliance With Hand Hygiene Guidelines: Experience From Tanzanian Labor Wards
Source: Glob Health Sci Pract. 2020 Dec 23;8(4):827–37. doi: 10.9745/GHSP-D-20-00221 (PMC7784080; doi:10.9745/GHSP-D-20-00221)
Supplement: 20-00221-Gon-Supplement2.pdf [file 20-00221-Gon-Supplement2.pdf]

Supplement to: Gon G, Ali SM, Aunger R, et al. A practical guide to using time-and-motion methods to monitor compliance with hand hygiene guidelines: experience from Tanzanian labor wards. *Glob Health Sci Pract*. 2020;8(4). https://doi.org/10.9745/GHSP-D-20-00221

Supplement 2. Reporting using STAMP

**Area and Element**	**Ref. Code**	**Description**
Intervention	INT.1–INT.3	Not applicable. This was an observational study with no interventions conducted.
Empirical setting		
Institution type	ES.1	The 10 health care facilities with the highest volume of deliveries ranging from 75 to 930 in Pemba and Unguja islands, Zanzibar, Tanzania. Eight of these facilities had an operating theater.
Care area	ES.2	Data collectors were observing any health care worker involved in the delivery process, hence they would usually be sitting in the labor room. If no deliveries were imminent, we asked data collectors to observe vaginal examinations in other rooms where they were conducted (e.g., the antenatal ward or examination room).
Locale	ES.3	Urban (6 facilities) and rural (3 facilities may be considered rural; 1 is definitely rural)
Research design		
Protocol	RD.1	Observational study
Duration	RD.2	3.5 months (September to December 2016)
Shift distribution	RD.3	Morning, afternoon, and night
Observation hours	RD.4	489:25:45 (hours:minutes:seconds)
Task category		
Definition and classification	TC.1	See Supplement 1 for definitions of tasks that included all birth attendants hand actions: procedures (i.e., patient–attendant interactions, such as a vaginal examination), hand hygiene or glove actions (hand rubbing/washing, drying, glove use, etc.), or some other touch (e.g., touching a pen or touching equipment).
Acknowledgement of prior work	TC.2	We used *Hand Hygiene Technical Reference Manual*,[7] *Hand Hygiene in Outpatient and Home-Based Care and Long-term Care Facilities*,[21] and *Pregnancy, Childbirth, Postpartum and Newborn Care: A Guide for Essential Practice*[22] to list the procedures (what we also call key attendant–patient interactions) that can occur during labor and delivery, and to list any other hand actions birth attendants can undertake before and after each of these procedures.
New development	TC.3	Not applicable as previous work described above.
Observer		
Size of field team	OBS.1	Three data collectors
Training	OBS.2	Training for this tool involved 3 days in the classroom using role-play (e.g., staged observation) and presentations. Each data collector also had a chance to practice the tool for 3 hours in the labor ward while being supervised by the trainer. In addition, the trainer carried out 2 hours of simultaneous observation with each of the data collectors and provided relevant feedback. Role-play in the classroom and exercises in the labor wards during the training also helped to add minor refinements to the tool.

Background	OBS.3	Data collectors (observers) were trained nurse-midwives working in managerial roles. Two of these data collectors worked in 2 of the study facilities but not in their labor wards. The third data collector worked in district-level management. Their previous knowledge and understanding of the process of labor were vital for the success of our project.
Interobserver uniformity	OBS.4	Details of the interobserver agreement are in the main manuscript. The kappa statistic calculated for pairs of data collectors was good for 2 of the 3 pairs at 93% and 90%, but was below the optimal level of 85% for one of the pairs, at 73%.[18]
Continuity	OBS.5	Not applicable. The 3 data collectors were the same throughout the study and each participated in observation in each of the 10 facilities.
Assignment	OBS	We consulted the ward rosters to allocate individual health workers to the observers, which was important because each birth attendant had a unique identifier that observers needed to input into WOMBAT when observing them. Shift allocation to data collectors was based on the following principles: (a) the same observer should observe the same attendant so the attendant becomes accustomed to the same person being on the ward; (b) the initial attendant–observer pairs at each facility were assigned at random (unless specific concerns were raised; e.g., some flexibility on choice of types of shifts was allowed to accommodate observers' needs); and (c) observation days should ideally be planned during changes in shift pattern to allow observation of the same attendants working on different types of shifts. The need to observe the same birth attendant across different shifts using the same observer had implications for the fieldwork duration and therefore had to be counterbalanced by our budget.<br><br>Because the allocation of responsibilities across birth attendants during a shift were usually decided during the shift itself, we encouraged observers to listen in on staff meetings on a daily basis to be aware of this allocation. This meant that an observer knew which birth attendant was most likely to perform the procedures outlined above (Table 1) that day and was able to decide whom to spend more time observing, also based on whom she observed the previous days. Indeed, observers were instructed that the aim was to observe each birth attendant roughly equally in each facility.
Subject		
Size	SUB.1	104 birth attendants were observed.
Recruitment and randomization	SUB.2	Data collectors were trained to observe all the allocated birth attendants that were involved in vaginal examinations and assisting deliveries.
Continuity	SUB.3	Not applicable. This study did not have multiple study phases.
Background	SUB.4	Birth attendants in our study were all women; 90% were professionally trained, and 10% were health orderlies/nonprofessionals.

Supplement to: Gon G, Ali SM, Aunger R, et al. A practical guide to using time-and-motion methods to monitor compliance with hand hygiene guidelines: experience from Tanzanian labor wards. *Glob Health Sci Pract*. 2020;8(4). https://doi.org/10.9745/GHSP-D-20-00221

Data recording		
Multitasking	DR.1	The hand actions were exhaustive (meaning that the list did not leave any possible actions out) and mutually exclusive (meaning that no 2 actions could occur simultaneously). We did not design a tool that aimed to capture multitasking or interruptions because we did not want to add to the burden of the data collectors.
Non observed periods	DR.2	We instructed observers to end a session when a major procedure was naturally over and no further patient activities were in sight, when the observer wanted to take a break, when there was the opportunity to start observing another birth attendant, or when the birth attendants would leave the room to perform other duties elsewhere.
Between task transitions	DR.3	Not applicable. No multiple tasks allowed.
Collection tool		WOMBAT
Data analysis		
Definition of key measures		The study aimed to capture hand hygiene compliance. All definitions required are highlighted in the manuscript and in Gon et al. 2018.
Analytical methods		STATA was used to analyze the data. We used descriptive statistics as well as logistic regression models accounting for individual level clustering (birth attendant level). Procedures are described in Gon et al. 2018.
Ancillary data		
Interruption	AD.1	Not applicable. Interruptions were not recorded.
Interaction	AD.2	The aim was to record all hand actions. Interpersonal communication was not recorded. We have information on whether the birth attendant touched her phone. No computers were available in the location of the observation in this study.
Location	AD.3	Data collectors were observing any health care worker involved in the delivery process, hence they would usually be sitting in the labor room. If no deliveries were imminent, we asked data collectors to observe vaginal examinations in other rooms where they were conducted (e.g., the antenatal ward or examination room).
