# Supplementary material for: A Practical Guide to Using Time-and-Motion Methods to Monitor Compliance With Hand Hygiene Guidelines: Experience From Tanzanian Labor Wards
Source: Glob Health Sci Pract. 2020 Dec 23;8(4):827–37. doi: 10.9745/GHSP-D-20-00221 (PMC7784080; doi:10.9745/GHSP-D-20-00221)
Supplement: 20-00221-Gon-Supplement1.pdf [file 20-00221-Gon-Supplement1.pdf]

Supplement to: Gon G, Ali SM, Aunger R, et al. A practical guide to using time-and-motion methods to monitor compliance with hand hygiene guidelines: experience from Tanzanian labor wards. *Glob Health Sci Pract*. 2020;8(4). https://doi.org/10.9745/GHSP-D-20-00221

Supplement 1. HANDS at Birth Tool

Room (room where you are sitting)

- Labor room
- Antenatal rom
- Vaginal examination (VE) room
- Other room

Context (availability of equipment and in-charge presence)

- Drying materials available
  - None
  - Single use
- Soap available
  - None
  - At the sink
  - Not near the sink
- Water available
  - None
  - Basin pour
  - Tap in room
  - Tap in next room
- Gloves available
  - None
  - In the room
- Alcohol handrub available
  - None
  - In the room
- In charge
  - Yes
  - No

Birth attendant (BA) status in room

- BA leaves
- BA enters

Woman

- 1
- 2
- other
  - 3
  - 4
  - 5
  - 6
  - 7
  - 8

    - 9
    - 10
    - 11
    - 12

Touched

- Macintosh/kanga (bed cover)
    - With body fluids
    - Clean
    - Not clean
    - Don't know
- Equipment (for delivery)
    - Sterile
    - With body fluids
    - After decontamination up to sterilization (any)
    - Don't know
- Other sterile/clean (e.g., cotton swabs)
- Body fluids–contaminated objects
    - Carry away placenta
    - Cloth for cleaning the bed
    - Other
- Objects (other)
    - Register, paper, or pen
    - Bag
    - Bin
    - Patient bed
    - Trolley
    - Tap
    - Phone
    - Mop or other cleaning material
    - Gloves pack
    - Own body
    - Other (unclean)
- Patient (woman or baby) (everything but inside legs and perineal area)

Delivery

- Delivery
    - Normal
    - Vacuum

- Kit
    - Premade or collected prior to delivery
    - Passed by colleague
    - Made along the way
    - Not seen

- Fast
  - Yes
  - No

- Workload
  - Working alone
  - 1 assistant
  - 2+ assistants

- Complicated (if you suspect the woman is having a complication, unless it is obvious, confirm with BA first)
  - No complications
  - Breech delivery: comes out with the buttocks or feet
  - Prolonged labor (ask BA): 12 hours for prima gravida, and 8 hours for multipara
  - Hemorrhage (antenatal): excessive blood loss (>500 mL); confirm with BA
  - Pre-eclampsia/eclampsia (ask BA): high blood pressure (>140/90 on 2 occasions or >160/110) plus either 1+ protein on urine dip or cerebral/visual disturbance (e.g., severe headache/reduced conscious level/blurred vision); or seizure attributable to pre-eclampsia (confirm with BA)
  - Baby floppy and blue
  - Premature rupture of membrane: ask the BA
  - Induction/augmentation: process to induce/speed up labor using drugs
  - Other (e.g., fever)
  - Not able to determine

Other procedures

- Vaginal examination
- Cord clamping and cutting
- Wiping/cleaning vagina
  - Clean material
  - Unclean material
  - Not seen
- Other
  - Catheter
  - Manual removal of placenta
  - Suturing
  - Intravenous (IV) fluids (insertion or removal)
  - IV blood line (insertion or removal)
  - Breaking amniotic membranes
  - Nasal suction (newborn)
  - Episiotomy
  - Newborn resuscitation—bag and mask
  - Changing IV bag
  - Adjusting IV line

    - Vital signs
    - Supporting breastfeeding
    - Injection
    - Wiping baby
- Cord traction
- Postdelivery exam (checking for tears)

Hand washing

- Duration
    - 1–9 seconds
    - ≥10 seconds
    - Not seen
- Soap
    - Yes
    - No
    - Not seen
- Behind fingers
    - Yes
    - No
    - Not seen

Drying

- Single use
- Other
    - Reusable
    - Own gown
    - Air
    - Other
    - Not seen
- None

Hand rubbing

- Duration
    - 1–9 seconds
    - ≥10 seconds
    - Not seen
- Behind fingers
    - Yes
    - No
    - Not seen

Gloves

- Action
    - Add
    - Remove
- Status
    - One hand

- Both hands
- Bare hands
